# Hints to Travelers

**Published:** 1877-08

**Authors:** 


### Hints for the Travelling Season.

At this season many persons contemplate travelling; to do so with the largest amount of comfort and advantage, physical, social, and mental, the following suggestions are made:

Take one fourth more money than your actual estimated expenses.

Acquaint yourself with the geography of the route and region of travel.

Have a good supply of small change, and have no bill or piece higher than ten dollars, that you may not take counterfeit change.

So arrange as to have but a single article of luggage to look after.

Dress substantially; better to be too hot for two or three hours at noon, than to be too cool for the remainder of the twenty-four.

Arrange, under all circumstances, to be at the place of starting fifteen or twenty minutes before the time, thus allowing for unavoidable or unanticipated detention on the way.

Do not commence a day's travel before breakfast, even if that has to be eaten at daylight. Dinner or supper, or both can be more healthfully dispensed with, than a good warm breakfast.

Put your purse and watch in your vest-pocket, and all under your pillow, and you will not be likely to leave either.

The most if not secure fastening of your chamber-door is a common bolt on the inside; if there is none, lock the door, turn the key so that it can be drawn partly out, and put the wash-basin under it; thus, any attempt to use a jimmy or put in another key, will push it out, and cause a racket among the crockery, which will be pretty certain to rouse the sleeper and rout the robber.

A sixpenny sandwich eaten leisurely in the cars, is better for you than a dollar dinner bolted at a "station."

Take with you a month's supply of patience, and always think thirteen times before you reply once to any supposed rudeness or insult, or inattention.

Do not suppose yourself specially and designedly neglected, if waiters at hotels do not bring what you call for in double quick time; nothing so distinctly marks the well bred man as a quiet waiting on such occasions; passion proves the puppy.

Do not allow yourself to converse in a tone loud enough to be heard by a person two or three seats from you; it is the mark of a boor if in a man, and of want of refinement and lady-like delicacy, if in a woman. A gentleman is not noisy; ladies are serene.

Comply cheerfully and gracefully with the customs of the conveyances in which you travel, and of the places where you stop.

Respect yourself by exhibiting the manners of a gentleman and a lady, if you wish to be treated as such, and then you will receive the respect of others.

Travel is a great leveller; take the position which others assign you from your conduct rather than from your pretensions.

